# Retinal biomarkers for predicting Alzheimer’s disease and Frontotemporal dementia: A prospective cohort study of 30573 participants

**DOI:** 10.1002/alz.088123

**Published:** 2025-01-09

**Authors:** Ruihan Wang

**Affiliations:** ^1^ West China Hospital of Sichuan University, Chengdu China

## Abstract

**Background:**

Alzheimer’s disease (AD) and Frontotemporal dementia (FTD) are the two most common causes of early‐onset dementia. Differentiating between AD and FTD can be challenging due to overlapping cognitive and behavioral manifestations. However, evidence on non‐invasive and early‐stage biomarkers for differentiating AD and FTD is still limited. Optical coherence tomography (OCT), as a non‐invasive retinal imaging technique, has provided enhanced access to detailed retinal neuronal structure. Our aim was to identify retinal biomarkers for predicting AD and FTD in non‐demented population and explore underlying brain structural mechanism.

**Method:**

We involved a total of 30573 UK Biobank participants without dementia, ocular disorders, and diabetes who underwent baseline retinal OCT imaging. Cox proportional hazard models were used to estimate the associations of macular OCT measurements with the risk of AD and FTD, adjusted for multiple confounding factors. Mediation models were applied to explore the underlying mechanisms driven by brain structures in subgroup participants with MRI scans.

**Result:**

The mean age at recruitment was 55.27, and 46.10% of the participants were male. During a mean follow‐up of 9.15 ± 2.59 years, 148 AD and 8 FTD patients were identified. Greater thickness of ganglion cell‐inner plexiform layer (GC‐IPL) was associated with reduced risk of AD (HR, 0.968; 95% CI, 0.938–0.999; p=0.044), while thicker retinal pigment epithelial (RPE) at inner superior subfield was associated with lower risks of FTD (HR, 0.710; 95% CI, 0.534–0.943; p=0.018). Decreased cortical (mainly including regions in occipital, temporal, and parietal cortices) and subcortical (thalamus and hippocampus) grey matter volume, and white matter integrity mediated the effects of GC‐IPL thickness on AD risk.

**Conclusion:**

Our findings provides evidence regarding the longitudinal association between macular OCT measurements and dementia. Thinning of the inner retinal layer is linked to a higher risk of AD, while thinning of the outer retinal layer is associated with an increased risk of FTD. Brain structures related to visual pathways mainly mediate the association between GC‐IPL and AD.

